# 

**Published:** 1889-04

**Authors:** 


					﻿JkFRIL, 1889,
OLD SERIES
Vol. xxv
NEW SERIES
Vol. VI -No. 2/
<7 1855 4
Wm»
inh:c.'L'/St <<
JOURNAL
i^ax et	hior by

$5^
W, F. WESTMORELAND, M. D.
H. V. M. MILLER, M. D. LL. D,
VIRGIL 0. HARDON, M. D.
Published By james p.HARRisoN&cd
ffi. ___________SST LANTA, GA.
SUBSCRIPTION 8 S2.0O A YEAR
				

